# Transition Metal Salts of Carboxylated Multiwalled Carbon Nanotubes in Combination with *N*-hydroxyphthalimide as Catalytic Systems for Hydrocarbon Oxidation

**DOI:** 10.3390/ma14092314

**Published:** 2021-04-29

**Authors:** Kamil Peckh, Beata Orlińska

**Affiliations:** Department of Organic Chemical Technology and Petrochemistry, Silesian University of Technology, B. Krzywoustego 4, 44-100 Gliwice, Poland; kamil.peckh@polsl.pl

**Keywords:** oxidation, carbon nanotubes, *N*-hydroxyphthalimide, ethylbenzene, oxygen

## Abstract

In this study, the transition metal (Co (II), Cu (II), and Mn (II)) salts of carboxylated carbon nanotubes were synthesized and characterized (the determined metal contents were in the range of 0.89–1.16%). The catalytic activity and the possibility for recovery and reuse of the obtained heterogeneous salts were then studied in the solvent-free oxidation of ethylbenzene with oxygen. The oxidation processes were carried out at 80 °C under atmospheric pressure in the presence of *N*-hydroxyphthalimide. The highest conversion of ethylbenzene, 27%, was obtained with a system consisting of the Cu (II) salt of the carboxylated carbon nanotubes, *N*-hydroxyphthalimide, and the azo initiator AIBN.

## 1. Introduction

The oxidation of hydrocarbons in the liquid phase, with the use of oxygen or air, allows valuable products to be obtained from cheap and simple petrochemical raw materials. These processes are used on a large scale to obtain, among others, terephthalic acid, cumene hydroperoxide, and mixtures of cyclohexanone and cyclohexanol (K/A oil) [1]. Another important process is the oxidation of ethylbenzene (EB) to hydroperoxide and/or acetophenone. The EB oxidation process is carried out in the temperature range of 130–150 °C, the conversion of the raw material is about 25%, and the selectivity for hydroperoxide is up to 85%. In order to obtain acetophenone (AcPO) as the main product, the oxidation process is often carried out with cobalt or manganese salts [2]. Ethylbenzene hydroperoxide is used in the industry as an oxidizing agent in the synthesis of propylene oxide. Acetophenone is used as an intermediate in the synthesis of, inter alia, perfumes, pharmaceuticals, and resins [3]. In the industry, acetophenone is obtained mainly as a co-product in the synthesis of phenol by the Hock method. Furthermore, it can be obtained by the acylation of benzene, but this method has no industrial significance.

The oxidation of ethylbenzene, as well as other alkylaromatic hydrocarbons, proceeds according to a free-radical chain mechanism. The compounds of transition metals such as salts of Cu (I)/Cu (II), Co (II)/(III), and Mn (II)/(III), for which the actions are mainly to accelerate the decomposition of the initially formed hydroperoxides, are often used as reaction catalysts. The resulting radicals initiate subsequent reaction chains, and the products are 1-phenylethanol and acetophenone. The catalytic activity of NHPI (*N*-hydroxyphthalimide) and NHPI systems with these transition metal compounds has also been described. Initially, Ishii used NHPI in the oxidation of ethylbenzene [4], which obtained a 34% yield of acetophenone (100 °C, 0.1 MPa). Under the same conditions and using an NHPI (10 mol%)/Co(acac)_2_ system (0.5 mol%), Ishii obtained a high conversion of ethylbenzene (91%) and a selectivity for AcPO of 93% [5]. In the EB oxidation process, complexes based on Fe(hemin) with NHPI have also been used [6], giving a AcPO selectivity of 94.3% and conversions exceeding 90% (100 °C, 0.3 MPa). A very high selectivity for AcPO (97%) was achieved with the use of oxobis (8-quinolinolato) vanadium (IV) complexes and NHPI. Under atmospheric pressure at a temperature of 90 °C and while using benzonitrile as a solvent, the conversion of ethylbenzene ranged from 60% to 69% [7]. The type of solvent used is also important. The authors of Reference [8] observed a much higher conversion of ethylbenzene (87.8%) using hexafluoropropan-2-ol than with the other solvents. They employed a catalyst system that consisted of Co(OAc)_2_ and NHPI at 30 °C and a pressure of 0.1 MPa.

Intensive work has also been carried out on catalysts for oxidation processes with oxygen, characterized not only by high selectivity and activity but also by the ease of isolation from post-reaction mixtures, which is particularly important during process commercialization [9]. Recent reports are concerned with the combination of NHPI with heterogeneous catalysts based on the mesoporous aluminosilicates on which phenanthroline (phen-MCM-41) had been immobilized [10]. The authors of this reference used a catalytic system that consisted of NHPI immobilized on the surface of silica with magnetic properties (Fe_3_O_4_ @ NHPI), phen-MCM-41, and Br_2_ to oxidize, with acetonitrile as solvent, various alkyl aromatic hydrocarbons, including ethylbenzene. A 97% conversion of the feed with 100% selectivity to acetophenone was achieved by a reaction at 80 °C. The authors of Reference [11] used a heterogeneous catalyst in the form of CuMgAl-LDH (layered double hydroxide) with the addition of NHPI, using benzonitrile as a solvent. In a reaction at atmospheric pressure and a temperature of 100 °C, the conversion rate of ethylbenzene was 99.3% with the selectivity to AcPO exceeding 97%.

Carbon nanotubes (CNTs) are of particular interest as carriers for the process of free radical oxidation with oxygen due to their high surface areas, tunable surface properties, superior mechanical strength, thermal stability, and low liability to corrosion. Due to their lack of micropores, active centers are concentrated on the surface, which increases their availability and ensures that the diffusion of reagents into the pores does not limit the process. We consider their catalytic activity in free radical oxidation processes to be a particularly interesting property compared to that in the other carriers.

**Figure 1:**
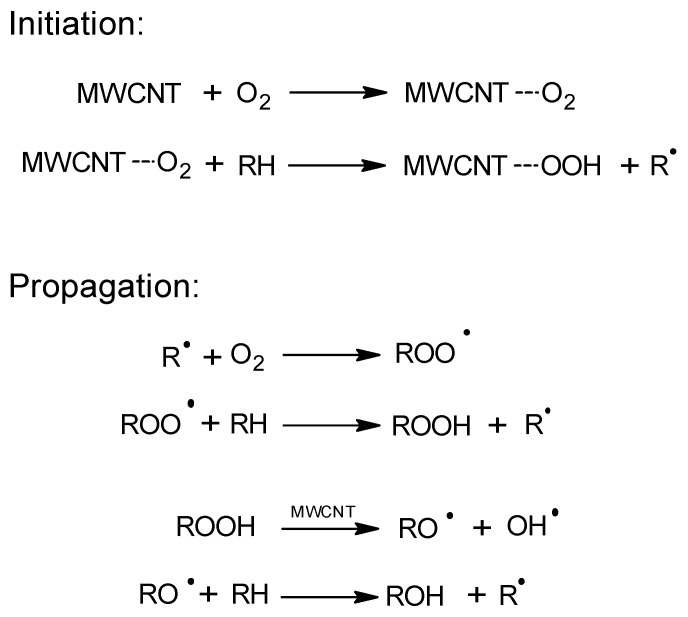
The free-radical oxidation mechanism on MWCNT (multiwalled carbon nanotubes).

The catalytic activity of carbon nanotubes in the EB oxidation process was described for the first time by Luo et al. [12]. These authors obtained a high conversion of the raw materials (38.2%) and a selectivity to AcPO of 60.9% (155 °C, 1.5 MPa). They proposed a process mechanism in which the catalytic effect of multiwalled carbon nanotubes (MWCNTs) resulted from acceleration of the decomposition of the originally formed hydroperoxide (Figure 1). The catalyst remained active for six cycles.

A similar activity of CNTs in the oxidation of cumene with oxygen was demonstrated by Liao et al. [13]. These authors oxidized cumene in the presence of commercial MWCNTs that achieved a conversion of 24% (80 °C, 0.1 MPa) and a selectivity for the hydroperoxide of 88%. In comparison, the cumene conversion in the reaction without MWCNTs was only 3%. The study demonstrated the radical nature of the process. In addition to the activity of MWCNTs in the hydroperoxide decomposition reaction, the authors also suggested that MWCNTs participate in the initiation of the process through the formation and subsequent decomposition of hydroperoxyl groups on the surface of nanotubes. It was possible to recycle the catalyst over five consecutive reaction cycles while maintaining its activity.

CNTs modified with heteroatoms have been used in the cyclohexane oxidation reaction [14], achieving high cyclohexane conversions of greater than 30%. For example, when the conversion was 34%, the selectivities of a mixture of cyclohexanol and cyclohexanone and of adipic acid were 40% and 52%, respectively. The possibility of recycling the catalyst, which was modified with nitrogen, was confirmed and achieved stable results with respect to the product selectivity in several subsequent reaction cycles. The activity of phosphorus-doped and boron-doped CNTs in this reaction was also noted [15]. Cao et al. synthesized CNTs by CVD (chemical vapor deposition method) from xylene by using dopants: anilines to introduce additional nitrogen atoms into the CNT structure, triethylborate for the introduction of boron atoms, and triphenylphosphine for phosphorus. The significant differences in CNT morphology were noted. The reactions were carried out under a pressure of 1.5 MPa at 120 °C, with a 10% conversion of cyclohexane giving a 31% selectivity to adipic acid (0% for the reaction without the catalyst).

The acid-functionalized CNTs have also been used as a metal catalyst support. One method was to soak the CNT with a solution of a suitable metal compound and then to dry it to evaporate the solvent. This method was used to prepare a catalyst, based on CNT and the Pt, Ru, and Cu compounds, for the aniline oxidation process with air [16]. The following metal salts were used: hydrogen hexachloro-platinum (IV) (H_2_PtCl_6_∙6H_2_O), ruthenium trichloride (RuCl_3_∙H_2_O), and copper (II) chloride (CuCl_2_∙2H_2_O). For the reaction at 200 °C and at 2 MPa pressure, a slight increase in aniline conversion was achieved after the addition of nonmetallic CNTs and a large increase for metal-modified CNTs. Conversions of more than 60% were achieved for the Pt and Cu compounds.

In the oxidation of ethylbenzene, nanotubes that were filled with nanowires made of iron atoms were used [17]. Nanomaterials with various Fe contents were synthesized and, in the oxidation reaction, resulted in up to 36.8% EB conversion. It was observed that the iron-modified nanomaterials exhibited magnetic properties, which enabled their separation by means of a magnetic field and was described in Reference [18].

The use of cobalt nanoparticles occluded in graphitic nitrogen-doped CNT hybrids (Co@N/CNT) as a catalyst for the oxidation of ethylbenzene has also been described [19], and they demonstrated much higher activity than unmodified CNTs. For example, the conversion of EB in the presence of CNT was 12%, and with Co@GNT, it was more than 60% under the same conditions (120 °C, 5 h, 0.1 MPa).

## 2. Results and Discussion

In this study, the catalytic activities of transition metal salts of carboxylated carbon nanotubes (MWCNT-COO-M), and systems composed of MWCNT-COO-M and NHPI were reported for the first time. MWCNT-COO-M was distinguished by a simple method of synthesis, which is important with regard to its potential application in industrial processes.

### 2.1. Synthesis and Characteristics of MWCNT-COO-M

In this study, cobalt (II), manganese (II), and copper (II) salts of carboxylated carbon nanotubes (MWCNT-COO-M) were synthesized. Commercial carboxylated multiwall carbon nanotubes (MWCNT-COOH) of technical purity were used as solid supports. The choice of MWCNT-COOH was justified by their competitive price, which may have a significant impact on their potential use on an industrial scale. The manufacturer declared the dimensions of the MWCNT-COOH as 20–40 nm in diameter and 10–30 µm in length. The content of the carboxyl groups in the MWCNT-COOH used was determined by titration at 1.00 mmol/g. The selected transition metal salts have often been used as catalysts for free radical oxidation of hydrocarbons in the liquid phase.

**Figure 2:**
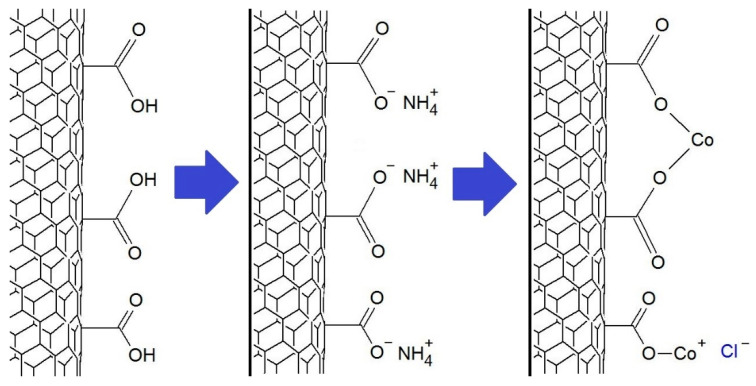
The scheme of the synthesis of MWCNT-COO-M using CoCl_2_ salt.

The immobilization of transition metal salts was carried out according to a previously described method [20]. The MWCNT-COOH reacted with an ammonia solution and, after filtration and drying, reacted with chloride solutions of the appropriate metals using ultrasound. The metal ions immobilized on the MWCNT-COOH surface may be bonded to one or two carboxyl groups, as shown in Figure 2.

**Figure 3:**
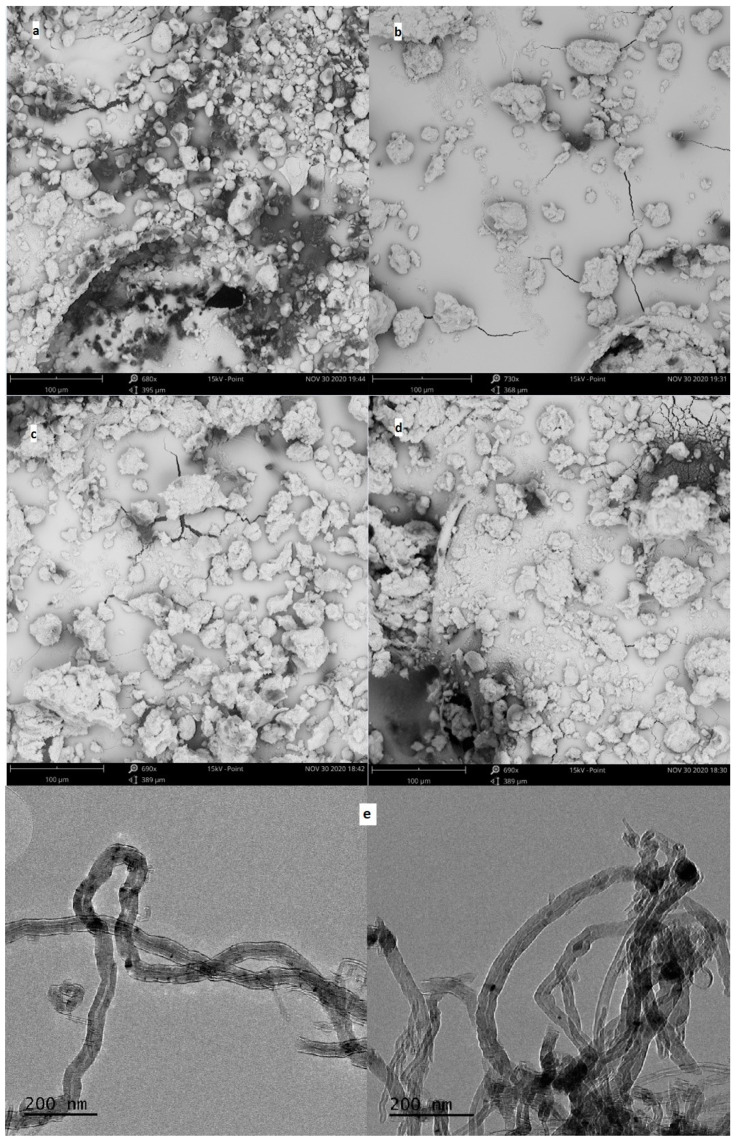
The SEM images of MWCNT powder for (**a**) MWCNT-COOH, (**b**) MWCNT-COO-Co, (**c**) MWCNT-COO-Mn, and (**d**) MWCNT-COO-Cu and (**e**) TEM images of MWCNT-COO-Co.

For the obtained MWCNT-COO-M, the content of the appropriate metal was determined, and SEM images as well as TEM images were taken (Figure 3). The determined metal contents were in the range of 0.89–1.16 wt%: 0.15, 0.17, and 0.21 mmol/g for MWCNT-COO-Co, MWCNT-COO-Cu, and MWCNT-COO-Mn, respectively. In the SEM images of the commercial MWCNT-COOH nanomaterial agglomerates, sizes in the range of 8–26 μm were mostly observed. In the nanomaterials after the immobilization process, much larger grains with dimensions from 48 to 100 μm as well as single nanotubes released from the agglomerates were observed. In the TEM images, we observed long (partially opened and broken) and tangled nanotube structures with diameters between 25 and 50 nm. The images suggest that most of the Co (II) particles are very small and irregularly dispersed on the outer surface of the CNT. Furthermore, CNT-synthesized catalytic nanoparticles occluded inside of the nanotubes can be seen.

### 2.2. Ethylbenzene Oxidation with MWCNT-COO-M

**Figure 4:**
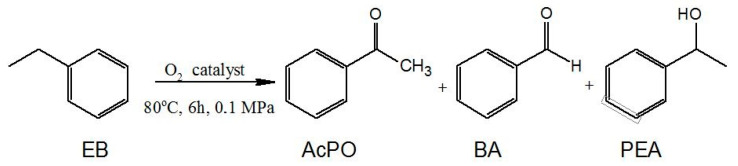
The ethylbenzene oxidation reaction.

The catalytic activity of the obtained salts for MWCNT-COO-M was tested in the oxidation of ethylbenzene with oxygen. The conversion of the raw material was determined on the basis of the amount of chemisorbed oxygen. The composition of the reaction mixture was determined by GC analysis, and the major oxidation products of ethylbenzene were found to be acetophenone (AcPO), 1-phenylethyl alcohol (PEA), and benzaldehyde (BA) (Figure 4). The presence of benzoic acid in the reaction products was observed only in a few reactions, but the selectivity did not exceed 0.4%. Apart from these compounds, 1-phenylethyl hydroperoxide (PEHP) was formed in minor amounts, as shown by the iodometric titration method.

Table 1 compares the EB conversion and the composition of the products obtained in the presence of the synthesized MWCNT-COO-M and the MWCNT-COO-M in combination with AIBN and/or NHPI. For the sake of comparison, reactions were also performed without MWCNT and with MWCNT-COOH used as a carrier (Table 1).

It was found that the addition of the carrier in the form of carboxylated carbon nanotubes MWCNT-COOH slightly increased the EB conversion from 1% to 4% (entries 1 and 5) or from 5% to 6% (entries 3 and 7) when it was used in combination with AIBN and NHPI.

The study showed the catalytic activities of the obtained Co (II), Cu (II), and Mn (II) salts of the carboxylated carbon nanotubes in EB oxidation. The highest EB conversions were obtained for the MWCNT-COO-M/NHPI/AIBN systems. They were significantly higher than what was obtained in the presence of the MWCNT-COOH support under similar conditions. The conversion increased depending on the metal salt used in the series, Mn (II) < Co (II) < Cu (II), from 13% to 27%. For comparison, when ethylbenzene was oxidized under similar conditions in the presence of a homogeneous Co(acac)_2_ (0.1 mol%), the conversion of 14.9% and the selectivity of acetophenone 74.9% were achieved [21].

It is known that the activity of transition metals results from the fact that they accelerate the decomposition of hydroperoxides. They may also have an influence on the generation of the PINO radical from NHPI.

The product produced in the greatest amount in the presence of MWCNT-COO-M/NHPI/AIBN systems was acetophenone, and its content in the products ranged from 66% to 69%, regardless of the immobilized metal ion.

**Figure 5:**
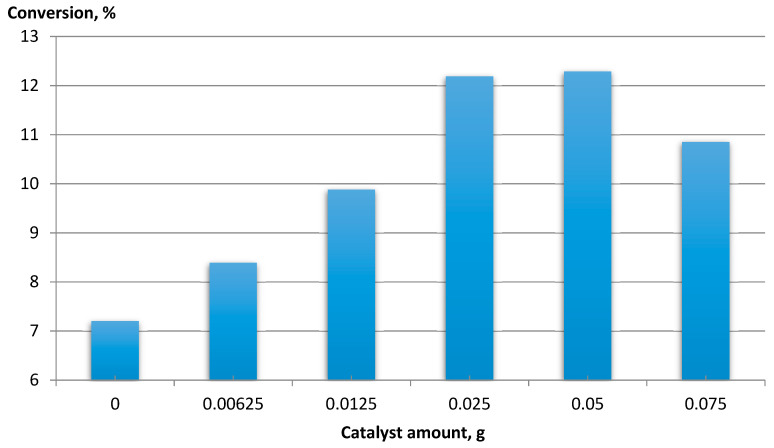
The dependence of EB conversion vs. the amount of catalyst. Ethylbenzene 16 mmol, NHPI 0.5 mol%, AIBN 0.2 mol%, 80 °C, 6 h, 1200 rpm, MWCNT-COO-Co.

For CNT-COO-Co, the effect of the catalyst amount on feed conversion was determined (Figure 5).

An increase in the conversion of the raw material was observed with an increase in the amount of catalyst to 0.025 g, while above 0.05 g, the conversion decreased. An increase in raw material conversion was observed with the increase in the amount of catalyst to 0.025 g. The conversion decreased above 0.05 g. This was due to the formation of a dense suspension, which made mixing difficult and thus limited the contact of oxygen with the reactants.

### 2.3. MWCNT-COO-M Recovery and Recycle

**Figure 6:**
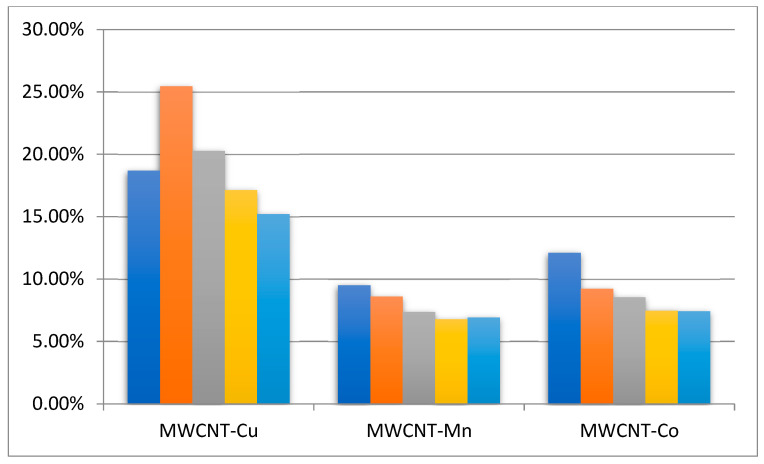
The conversion of ethylbenzene in subsequent reaction cycles. Ethylbenzene 80 mmol, NHPI 0.5 mol%, 80 °C, 6 h, 1200 rpm, MWCNT-COO-M cycle 0: 0.125 g.

An investigation into the possibility of the recovery and reuse of MWCNT-COO-M was carried out. The scale of the reaction was increased from 2 to 10 mL, and after the completion of the reaction, the MWCNT-COO-M was filtered off, washed with ethanol, and (after drying) reused for another four reaction cycles, adding new portions of NHPI and AIBN for each cycle. The EB conversion in subsequent cycles is shown in Figure 6, and the selectivity of the individual products is summarized in Table 2.

It was observed that, after increasing the scale of the reaction from 2 to 10 mL, the rate of reaction slightly decreased. This effect suggested that contact between oxygen and the reagents was slightly worse. The comparison of the conversion achieved in each cycle showed that, after five reaction cycles, the MWCNT-COO-Cu still demonstrated high activity. Unfortunately, the catalytic activity of MWCNT-COO-Co and MWCNT-COO-Mn decreased in the first and second recycles, but in the third and fourth recycles, it remained at a constant level higher than in the reaction using the MWCNT-COOH/NHPI/AIBN system. Tests were also undertaken to determine the possibility of leaching the metal into the reaction mixture during the process. For this purpose, after 1 h of reaction with the system containing 80 mmol of ethylbenzene and the MWCNT-COO-M catalyst, 2 mL of filtrate was taken and subjected to further oxidation. For all of the metal salts, no further oxygen chemisorption was observed during the 2 h reaction. This effect could indicate a stable binding of metal ions to MWCNT-COOH. It was further confirmed by determination of the metal content in the filtrates. The contents of Co and Mn in the filtrate were below 0.25 ppm (too low for quantitative determination), and the content of Cu was 0.44 ppm. This means that only traces of metal were washed away (0.19–0.27%), which should not cause the observed decrease in conversion. The decrease in catalyst activity may result, for example, from the adsorption of reagents on the CNT surface, which blocks access to the active sites.

## 3. Summary

The presented studies are in line with current research trends in the field of catalysis, i.e., the use of easily separable, durable, and highly active catalysts that enable multiple uses, and is in line with the tendency to develop oxidation processes using mild conditions with low pressure and temperature. The high activity of new catalytic systems consisting of transition metal salts of carboxylated multiwalled carbon nanotubes, NHPI, and AIBN was demonstrated. MWCNT-COO-M are distinguished by a simple method of synthesis and the use of technical purity and are supported by low prices, which are important with regard to its potential application in industrial processes. The highest conversion of EB (27%) was obtained when the MWCNT-COO-Cu/NHPI/AIBN system was used. After five reaction cycles, MWCNT-COO-Cu still demonstrated high catalytic activity.

The obtained MWCNT-COO-M are recoverable and recyclable and demonstrated high activity in EB oxidation reactions carried out in the presence of small amounts of NHPI (0.5 mol%) under mild solvent-free conditions (80 °C, 0.1 MPa, 6 h). Although a higher EB conversion could be achieved, these processes required homogeneous conditions or the use of significant amounts of NHPI (≥10 mol%) and polar solvents or required higher temperature and pressure. For example, the EB conversion was 93%, but a homogeneous system of NHPI (10 mol%) and Co(acac)_2_ in benzonitrile (2.5 mL/1 mmol EB) was used (100 °C, 6 h) [5]. The EB conversion was also high (82%) in the reaction performed in the presence of solid CuMgAl-LDH, but larger amounts of NHPI (10 mol%) and benzonitrile as solvents (2 mL/1 mmol of EB) were required (80 °C, 0.1 MPa, 3 h) [11]. Similarly, Faraji et al. oxidized ethylbenzene using Co (II) immobilized on silica and 15 mol% of NHPI in AcOH as a solvent (2.5 mL/1 mmol of EB) (100 °C, 8 h, conversion 87%) [22]. The authors of [23] used Co_3_O_4_ nanoparticles dispersed on reduced graphene oxide in combination with NHPI (6 mol%) in MeCN as a solvent (1:2, *v*/*v*) at higher temperatures and under the pressure of oxygen (120 °C, 0.3 MPa, conversion 84%).

## 4. Materials and Methods

### 4.1. Materials

The following compounds were used as-received: multiwalled carbon nanotubes with carboxylic functional groups (MWCNT-COOH, 95% Cheaptubes, Grafton, VT, USA), ethylbenzene (99% Acros Organics), CoCl_2_ (POCH, Gliwice, Poland), CuCl_2_ (POCH, Gliwice, Poland), MnCl_2_ (POCH, Gliwice, Poland), N-hydroxyphthalimide (NHPI, Sigma-Aldrich, Steinheim, DE, USA), NH_3_ 25 % solution (Chempur, Piekary Śląskie, Poland).

### 4.2. MWCNT-COO-M Synthesis

MWCNT-COOH (1.00 g) was added to 40 cm^3^ of 10% aqueous ammonia solution and sonicated for 3 h. After filtration and drying at 90 °C, 0.97 g of MWCNT-COONH_4_ was isolated.

MWCNT-COONH_4_ (0.2184 g) was added to 25 cm^3^ of solvent (water, acetone, or ethanol). A volume of 5 cm^3^ of 0.3 M of metal salt solution (CoCl_2_, MnCl_2_, or CuCl_2_) was added. The mixture was sonicated for 1.5 h. After filtration and 90 °C drying, 0.2040 g of MWCNT-COO-M was isolated.

### 4.3. Ethylbenzene Oxidation

The oxidation reactions were performed in a gasometric apparatus that was described previously [24] under atmospheric pressure. The apparatus was equipped with a burette, which enabled the determination of oxygen consumption under atmospheric pressure during the reaction and, thus, the determination of the feedstock conversion. The reactions were carried out at 80 °C for 6 h using 2 mL of ethylbenzene (16 mmol).

### 4.4. Catalyst Recovery and Recycle

In order to test the stability and the possibility of catalyst recycling, five consecutive reaction cycles were carried out with previously prepared nanotubes (MWCNT-COO-Co, MWCNT-COO-Mn, and MWCNT-COO-Cu). The reactions were conducted as described in Section 4.3. In order to reduce the inaccuracy due to the small scale, the ethylbenzene levels were increased to 10 mL (80 mmol). Samples were taken each time for iodometric and chromatographic analysis. The solution was then filtered, and the resulting precipitate was washed with 10 mL of ethanol and dried at 90 °C. The precipitate was then used in the next reaction. Initially, 0.125 g of MWCNT modified with the corresponding salts was used.

### 4.5. Iodometric Analysis

An iodometric analysis method was used to determine the presence of ethylbenzene hydroperoxide in the reaction mixture [25]. After the oxidation process, a sample of about 0.2 g (with an accuracy up to 1 × 10^−4^ g) was taken from the reaction mixture, and then 20 cm^3^ of glacial acetic acid was added and flushed with nitrogen. Then, each time, about 2 g of sodium iodide was added to the mixture. The flask was left in the dark for 30 min until iodine formed. After the appointed time, 20 cm^3^ of distilled water was added to the solution, and then, it was titrated with 0.1 M standard sodium thiosulfate solution using the Brand Digital Burette II with a 25 mL measuring range (subdivision 0.01 mL). The content of ethyl hydroperoxide in the obtained reaction mixture was too low, ≤1%, for quantitative analysis.

### 4.6. GC Analysis

After the oxidation process, a sample was taken from the reaction mixture and triethylphosphite (EtO)_3_P was added to reduce ethylbenzene hydroperoxide quantitatively to 1-phenylethanol. The amounts of PEA, BA, and AcPO were determined via GC analysis and used for calculation of the reaction’s selectivity. The analysis was carried out under the following conditions: a dosing temperature of 280 °C, column flow at 40 mL/s, split 1:100, injection volume of 0.5 μL, and a detector temperature of 320 °C. The analysis was carried out at an initial column temperature of 120 °C, which was held for 2.4 min. The column was then heated to 300 °C and then held at that temperature for 6 min, giving an overall analysis time of around 8 min. Acetonitrile was used as a solvent. The following standards were used in the analysis: benzoic acid, 1-phenylethanol, benzaldehyde, acetophenone, and naphthalene as an internal standard.

### 4.7. Apparatus

The morphologies of the carrier and MWCNT-COO-M were examined with the use of a Scanning Electron Microscope (SEM)—TM3000 TableTop of Hitachi High-Technologies Corporation brand (Tokyo, Japan) and a Transmission Electron Microscope (TEM)—TecnaiG2 F20 X-TWIN of ThermoFisher Scientific (Waltham, MA, USA). The GC analyses were performed using a Shimadzu (Kyoto, Japan) GC-2010 Plus gas chromatograph that was equipped with an FID detector and a ZB-5HT column with a length of 29.5 m and an internal diameter of 0.25 mm.

## Figures and Tables

**Table 1:** The conversion of EB and selectivity of oxidation products with MWCNT catalysts and NHPI.

Entry	Catalyst Type	AIBN, mol%	NHPI, mol%	Conversion, %	Selectivity, %		
					PEA ^a^	BA	AcPO
1	-	-	-	1	56	3	41
2		0.2	-	5	32	15	53
3		0.2	0.5	5	24	19	57
4		-	0.5	5	34	19	47
5	MWCNT-COOH	-	-	4	37	3	60
6		0.2	-	6	37	16	47
7		0.2	0.5	6	17	18	65
8	MWCNT-COO-Co	-	-	4	33	6	59
9		0.2	-	8	25	4	71
10		0.2	0.5	18	30	1	69
11	MWCNT-COO-Cu	-	-	3	35	3	62
12		0.2	-	8	25	3	72
13		0.2	0.5	27	28	6	66
14	MWCNT-COO-Mn	-	-	2	28	8	64
15		0.2	-	8	43	4	53
16		0.2	0.5	13	27	7	66

Ethylbenzene 16 mmol, NHPI 0.5 mol%, 80 °C, 6 h, 1200 rpm, MWCNT 0.025 g; ^a^ involves the formation of minor amounts of hydroperoxide.

**Table 2:** The selectivity of oxidation products with MWCNT catalyst recycling.

Entry	Cycle	Catalyst Type	Conversion, %	Selectivity, %		
				PEA ^a^	BA	AcPO
1	**0**	MWCNT-COO-Cu	19	26	4	70
2	**I**	MWCNT-COO-Cu	25	28	6	66
3	**II**	MWCNT-COO-Cu	20	30	18	52
4	**III**	MWCNT-COO-Cu	17	23	8	69
5	**IV**	MWCNT-COO-Cu	15	28	8	64
1	**0**	MWCNT-COO-Co	12	38	4	58
2	**I**	MWCNT-COO-Co	9	38	8	55
3	**II**	MWCNT-COO-Co	9	26	10	64
4	**III**	MWCNT-COO-Co	8	18	11	71
5	**IV**	MWCNT-COO-Co	7	31	8	61
1	**0**	MWCNT-COO-Mn	11	33	13	54
2	**I**	MWCNT-COO-Mn	10	32	15	53
3	**II**	MWCNT-COO-Mn	8	26	6	68
4	**III**	MWCNT-COO-Mn	7	30	19	51
5	**IV**	MWCNT-COO-Mn	7	30	20	50

Ethylbenzene 80 mmol, NHPI 0.5 mol%, AIBN 0.2 mol%, 80 °C, 6 h, 1200 rpm, MWCNT-COO-M cycle 0: 0.125 g. ^a^ Involves the formation of minor amounts of hydroperoxide.

## Data Availability

Data sharing not applicable.
